# Supplementary material for: Link between the numbers of particles and variants founding new HIV-1 infections depends on the timing of transmission
Source: Virus Evol. 2019 Jan 30;5(1):vey038. doi: 10.1093/ve/vey038 (PMC6354028; doi:10.1093/ve/vey038)
Supplement: Supplementary Data [file vey038_supp.zip › supplementaryFigures.pdf]

Link between the numbers of particles and variants founding new HIV-1 infections depends on the timing of transmission

Supporting Figures

R.N. Thompson[1,2,3,*], C. Wymant[4], R.A. Spriggs[5], J. Raghwani[1,4], C. Fraser[4] and K.A. Lythgoe[1,4,*]

[1]Department of Zoology, University of Oxford, South Parks Road, Oxford OX1 3PS, UK

[2]Mathematical Institute, University of Oxford, Andrew Wiles Building, Radcliffe Observatory Quarter, Oxford OX2 6GG, UK

[3]Christ Church, University of Oxford, St Aldates, Oxford OX1 3DP, UK

[4]Big Data Institute, Li Ka Shing Centre for Health Information and Discovery, Nuffield Department of Medicine, University of Oxford, Oxford OX3 7FZ, UK

[5]Department of Plant Sciences, University of Cambridge, Downing Street, Cambridge CB2 3EA, UK

*Correspondence to: robin.thompson@chch.ox.ac.uk, katrina.lythgoe@bdi.ox.ac.uk

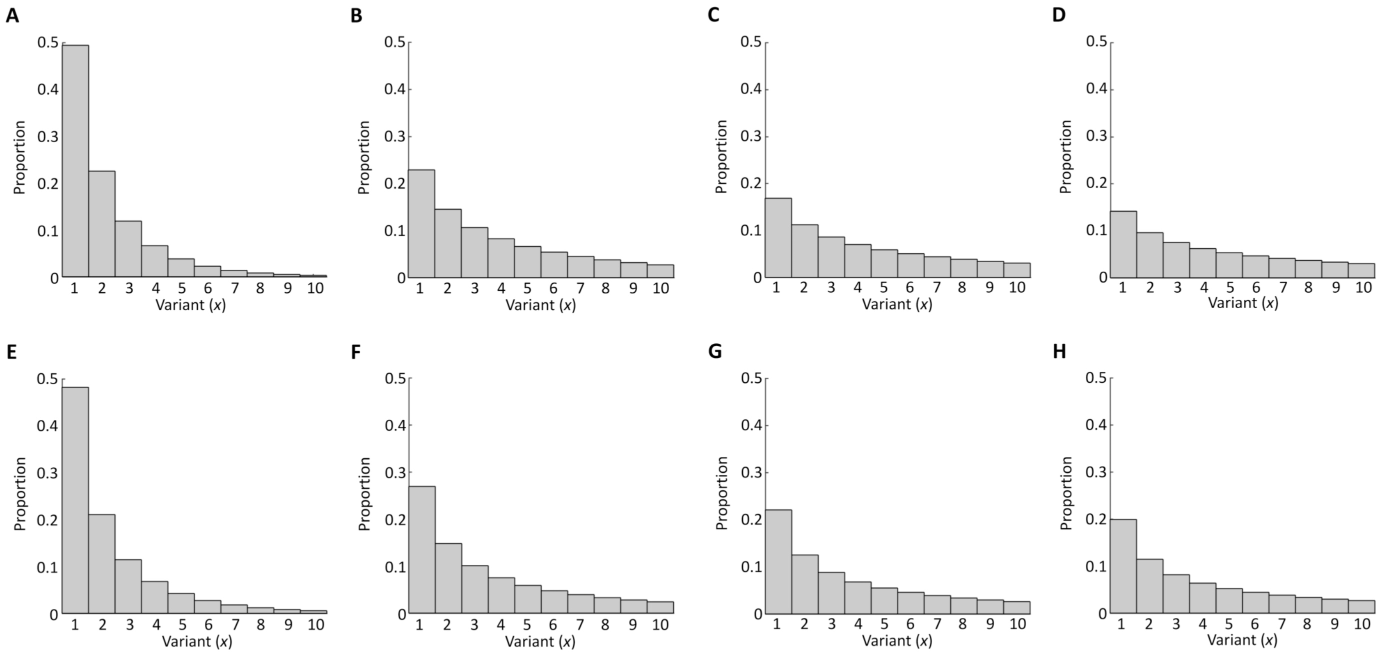

Figure S1. The distributions of variants in donors throughout their course of infection parameterised using data from p24 and nef. The best model fit is shown for p24 after: (A) 1 year; (B) 4 years; (C) 7 years; (D) 10 years. The best model fit is shown for nef after: (E) 1 year; (F) 4 years; (G) 7 years; (H) 10 years. The *x*-axis represents the xth most common variant at the time of sampling. The best fitting models and parameter values are given in Table 1 of Text S1.

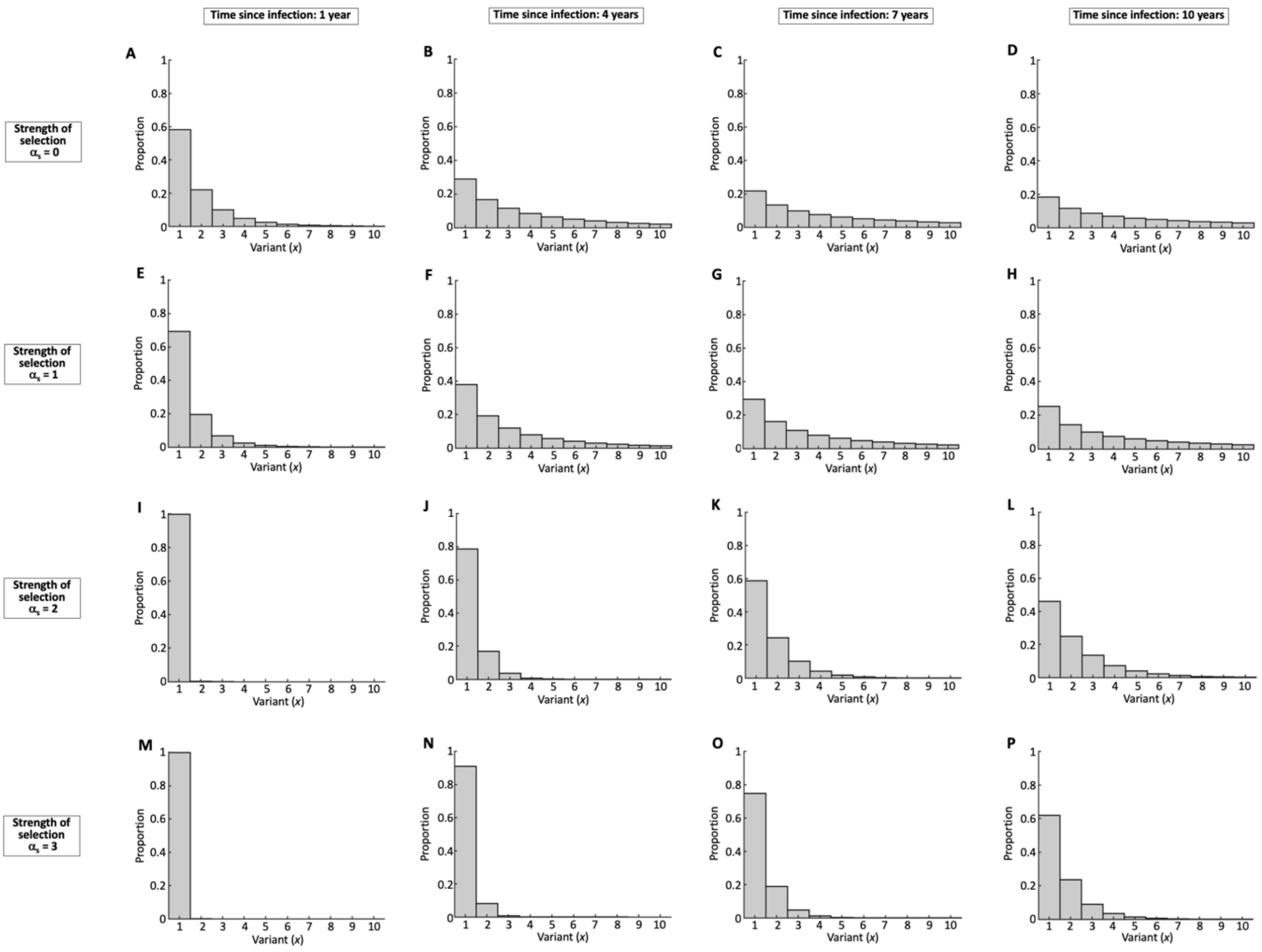

Figure S2. The distribution of variants throughout infection, for different assumed strengths of selection (α_s), using data from integrase. The best model fit is shown after 1, 4, 7 and 10 years for (A)-(D): $\alpha_s = 0$; (E)-(H): $\alpha_s = 1$; (I)-(L): $\alpha_s = 2$; (M)-(P): $\alpha_s = 3$. The *x*-axis represents the xth most common variant at the time of sampling after adjusting for selection (see Materials and Methods). The best fitting models and parameter values are given in Table 1 of Text S1.

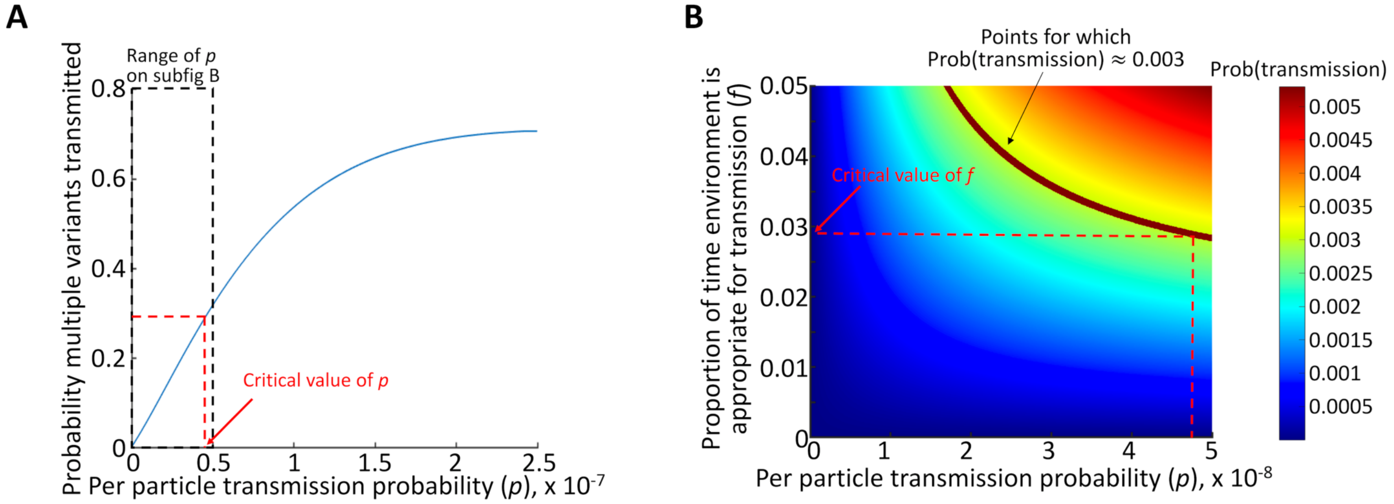

Figure S3. Parameterising the model. (A) The value of the per-act transmission probability (*p*) is chosen so that the probability of transmitting multiple variants in a single act conditional on transmission is 0.3; (B) The value of the proportion of the time the environment in the recipient is appropriate for transmission (*f*) is then chosen so that the probability of transmission per act is 0.003 at the value of *p* selected in panel A. Here this process gives the values $p = 4.715 \times 10^{-8}$ and $f = 0.029$. The case shown here is for no selection at transmission and no bias towards early infection. Where such a pair exists, there is always a unique pair of values *p* and *f* corresponding to Prob(multi-variant transmission) = 0.3 and Prob(transmission) = 0.003.

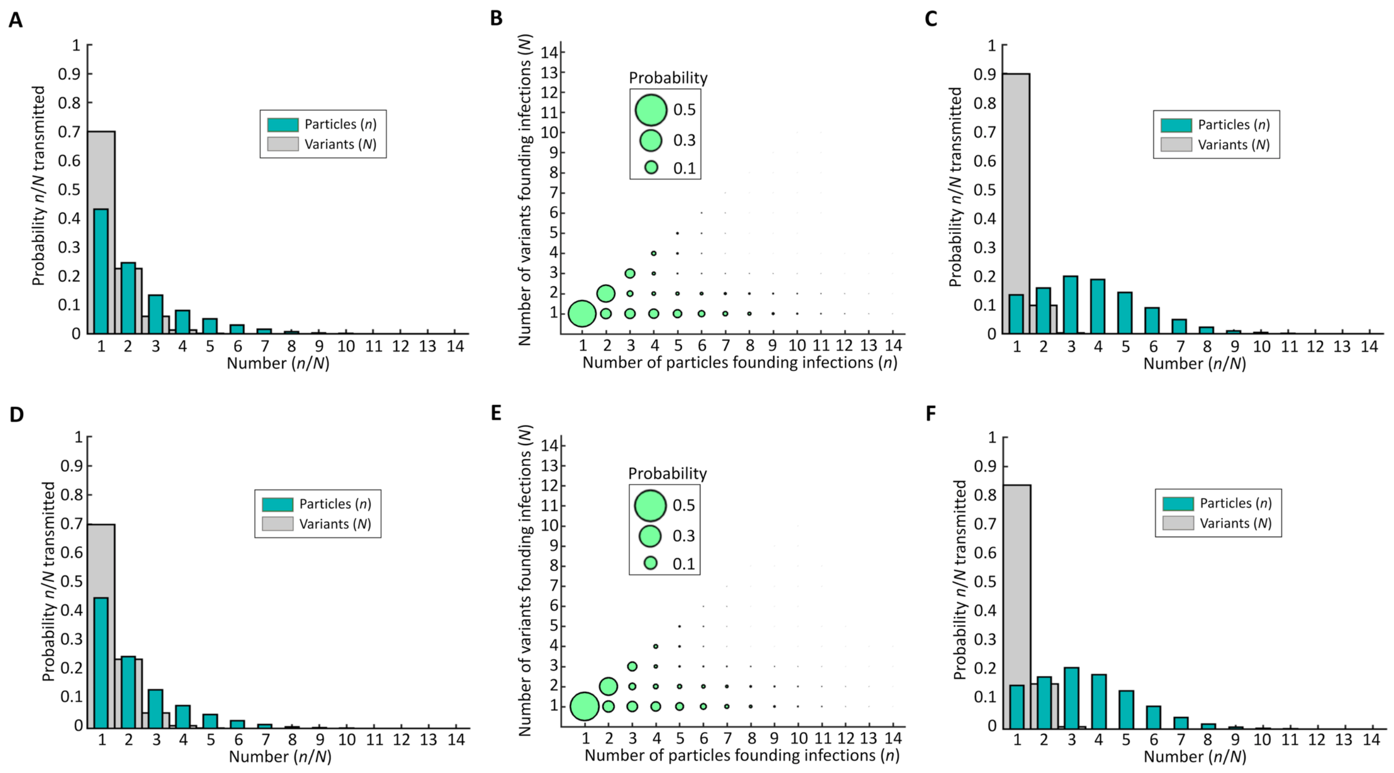

Figure S4. The qualitative results of our analyses are unchanged when our model is parameterised using sequencing data from other regions of the viral genome. (A) The distributions of the numbers of particles (teal) and numbers of distinct variants (grey) founding new infections in the population when the model is parameterised using sequencing data from p24. (B) The joint distribution of the numbers of particles and variants founding new infections when the model is parameterised using sequencing data from p24. The circle areas are proportional to the probabilities that they represent. (C) The distributions of the numbers of particles (teal) and numbers of distinct variants (grey) founding new infections in the population, from donors in early infection only (infected for less than two years), when the model is parameterised using sequencing data from p24. (D)-(F) Same as A-C but using sequencing data from nef. Parameter values: variant distribution parameter values are given in Table 1 of Text S1, and transmission parameter values are given in Table 2 of Text S1.

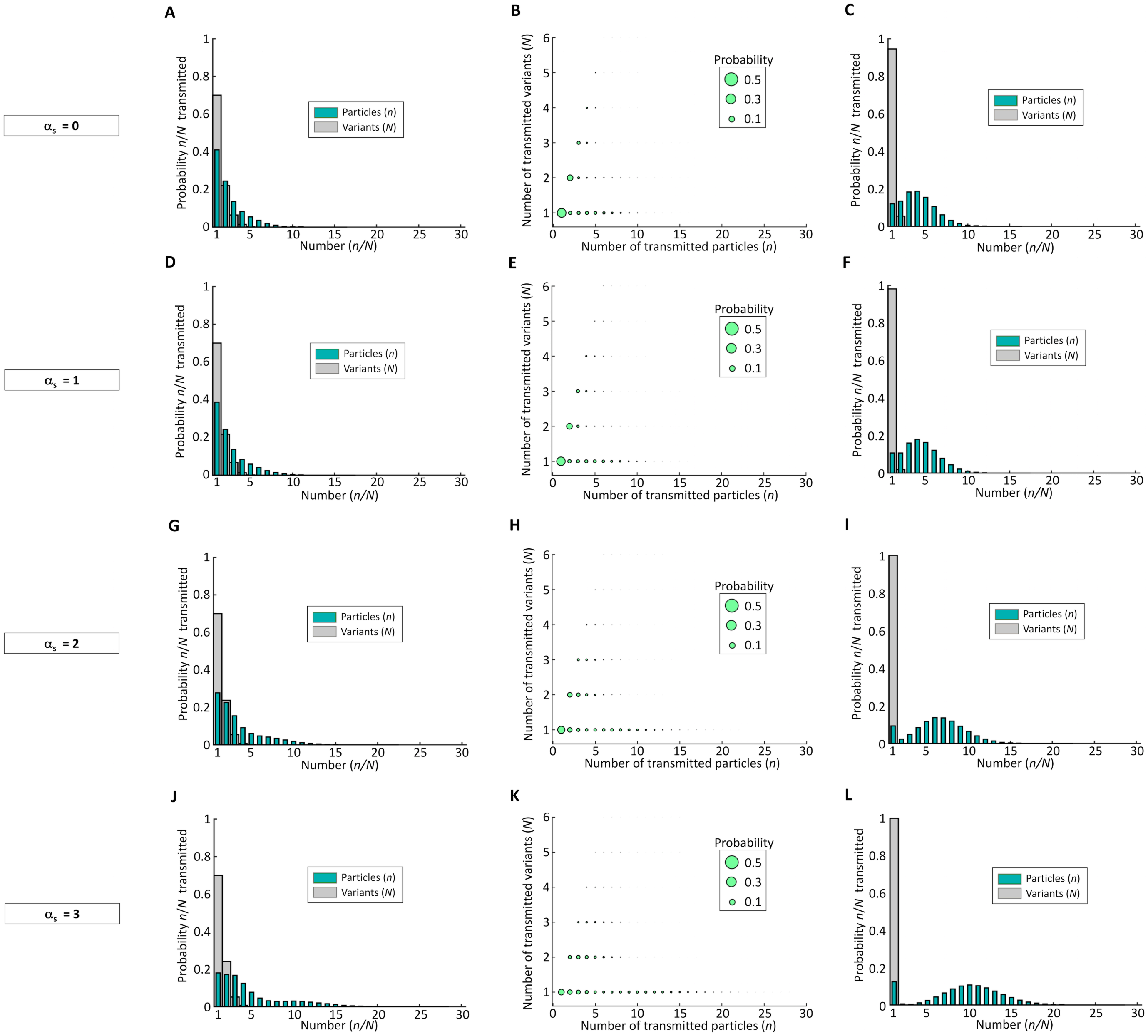

Figure S5. The impact of selection on the numbers of particles and viral variants that found new infections in the population. (A) The distributions of the numbers of particles (teal) and numbers of distinct variants (grey) founding new infections in the population with no selection ($\alpha_s = 0$). (B) The joint distribution of the numbers of particles and variants founding new infections with no selection ($\alpha_s = 0$). Circle areas are proportional to the probabilities that they represent. (C) The distributions of the numbers of particles (teal) and numbers of distinct variants (grey) founding new infections in the population, from donors in early infection only (infected for less than two years), with no selection ($\alpha_s = 0$). Panels D-F are the analogous results to A-C but with weak selection ($\alpha_s = 1$). Panels G-I are the analogous results to A-C but with strong selection ($\alpha_s = 2$). Panels J-L are the analogous results to A-C but with very strong selection ($\alpha_s = 3$). Parameter values: variant distribution parameter values are given in Table 1 of Text S1, and transmission parameter values are given in Table 2 of Text S1.

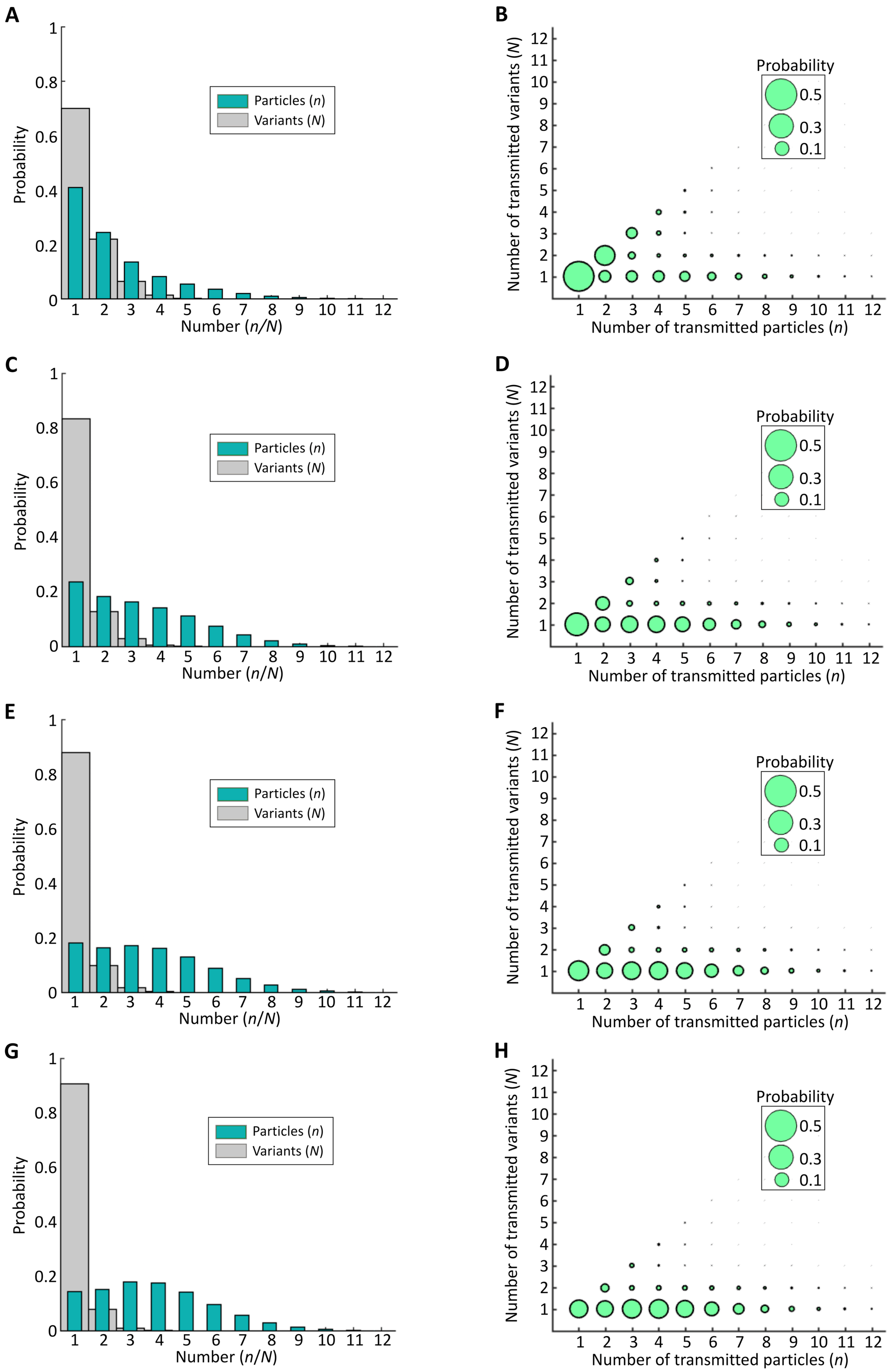

Figure S6. The distributions of the numbers of transmitted particles and variants when transmission is weighted towards early infection. Left column: The distributions of the numbers of particles (teal) and numbers of distinct variants (grey) founding new infections in the population. Right column: The joint distribution of the numbers of

particles and variants founding new infections. (A) and (B) No weighting towards early infection (*w* = 1). (C) and (D) Moderate weighting towards infections founded by donor in early infection (*w* = 5). (E) and (F) Strong weighting (*w* = 10). (G) and (H) Very strong weighting (*w* = 20). Parameter values: variant distribution parameter values are given in Table 1 of Text S1, and transmission parameter values are given in Table 2 of Text S1.

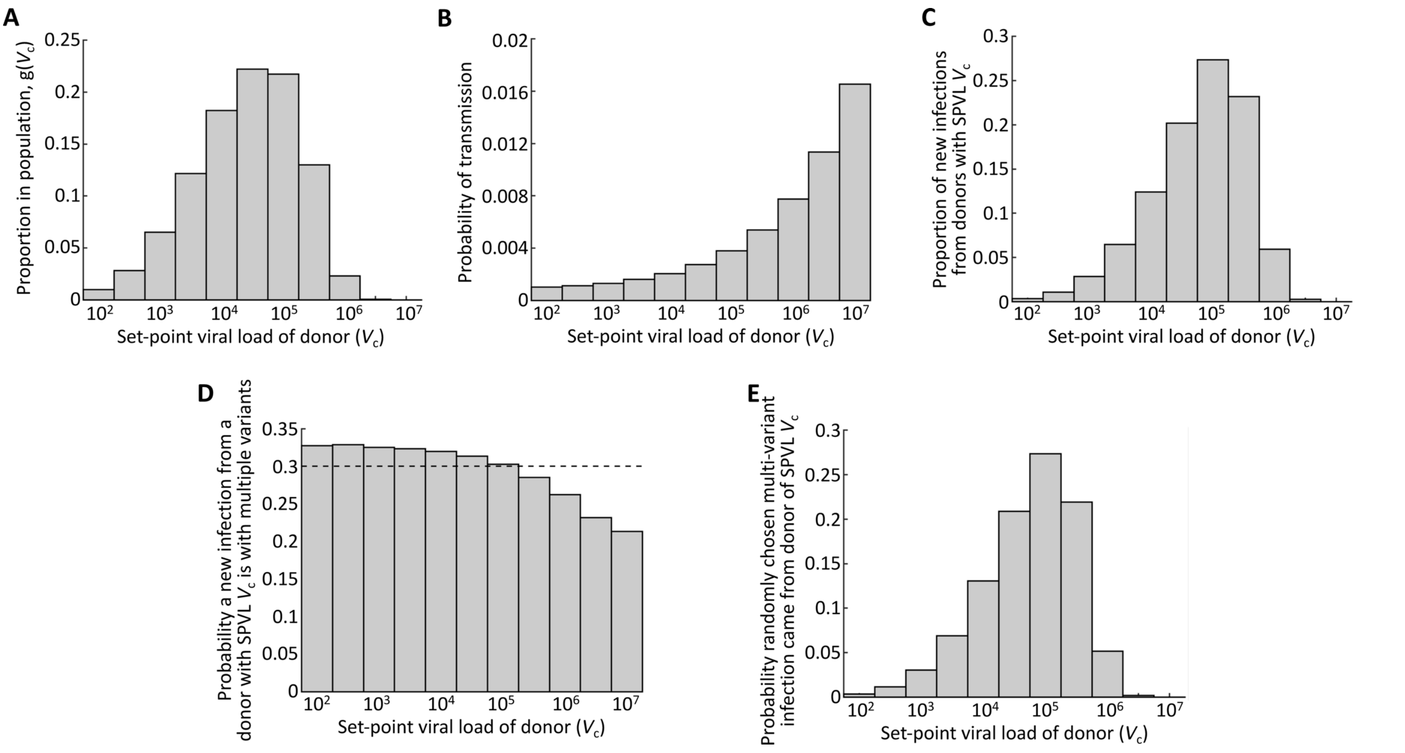

Figure S7. Determining which transmissions arise from individuals with different SPVLs. (A) The proportion of donors with each SPVL in the population. (B) The probability that a randomly chosen potential transmission act leads to transmission. (C) The proportion of new infections in the population arising from donors with each SPVL, evaluated as the normalised product of A and B. (D) Conditional on transmission, the probability that a new infection from a donor is with multiple variants. The dotted line represents the population average value. (E) The probability, for a randomly chosen new multi-variant infection in the population, that it arose from an individual with each set-point viral load, evaluated as the normalised product of C and D. Parameter values: variant distribution parameter values are given in Table 1 of Text S1, and transmission parameter values are given in Table 2 of Text S1.

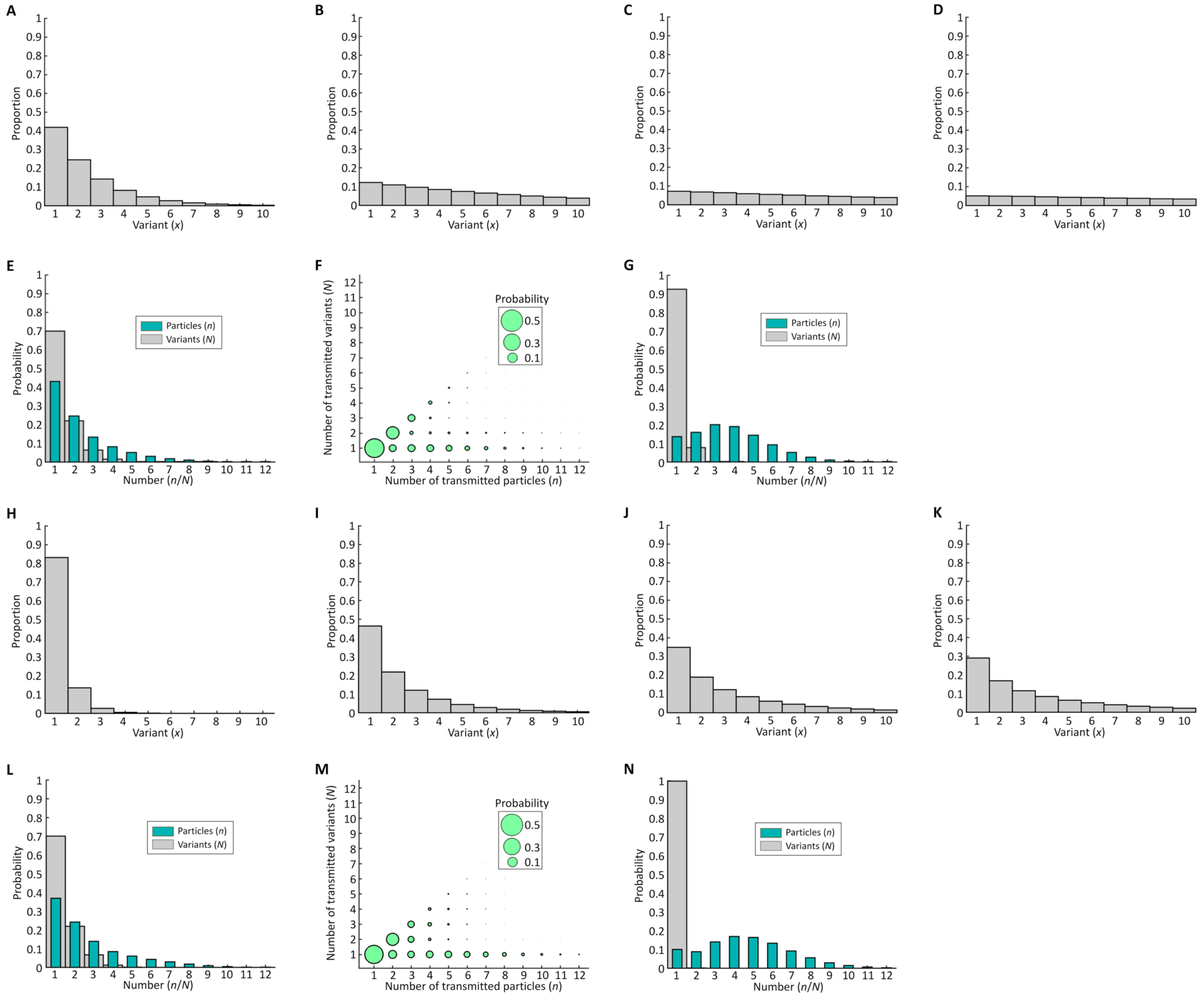

Figure S8. The distributions of the numbers of transmitted particles and variants for different variant diversity distributions within donors. Panels (A)-(D) show the distribution of variants in donors with high variant diversity who have been infected for 1 year, 4 years, 7 years and 10 years, respectively. The x-axis, representing the xth most common variant, is truncated after the tenth most common variant, but the full distribution is used in our model. (E) The distributions of the numbers of particles (teal) and numbers of distinct variants (grey) founding new infections in the population from donors with high variant diversity. (F) The joint distribution of the numbers of particles and variants founding new infections from donors with high variant diversity. (G) The distributions of the numbers of particles (teal) and numbers of distinct variants (grey) founding new infections in the population, from donors in early infection only (infected for less than two years), from donors with high variant diversity. Panels H-N are analogous to A-G but for donors with low variant diversity. To consider donors with different variant diversity, the parameters of the gamma distribution characterising variant diversity in the no selection and no bias towards early infection case (see Table 1 of Text S1) are multiplied by appropriate factors. For high diversity the parameter δ is multiplied by factor 2.5 (so that δ = 1.043), and for low diversity the parameter η is multiplied by factor 2.5 (so that η = 1.41). The transmission parameter values are then reparameterised to fit the population-level data (see Table 2 of Text S1).

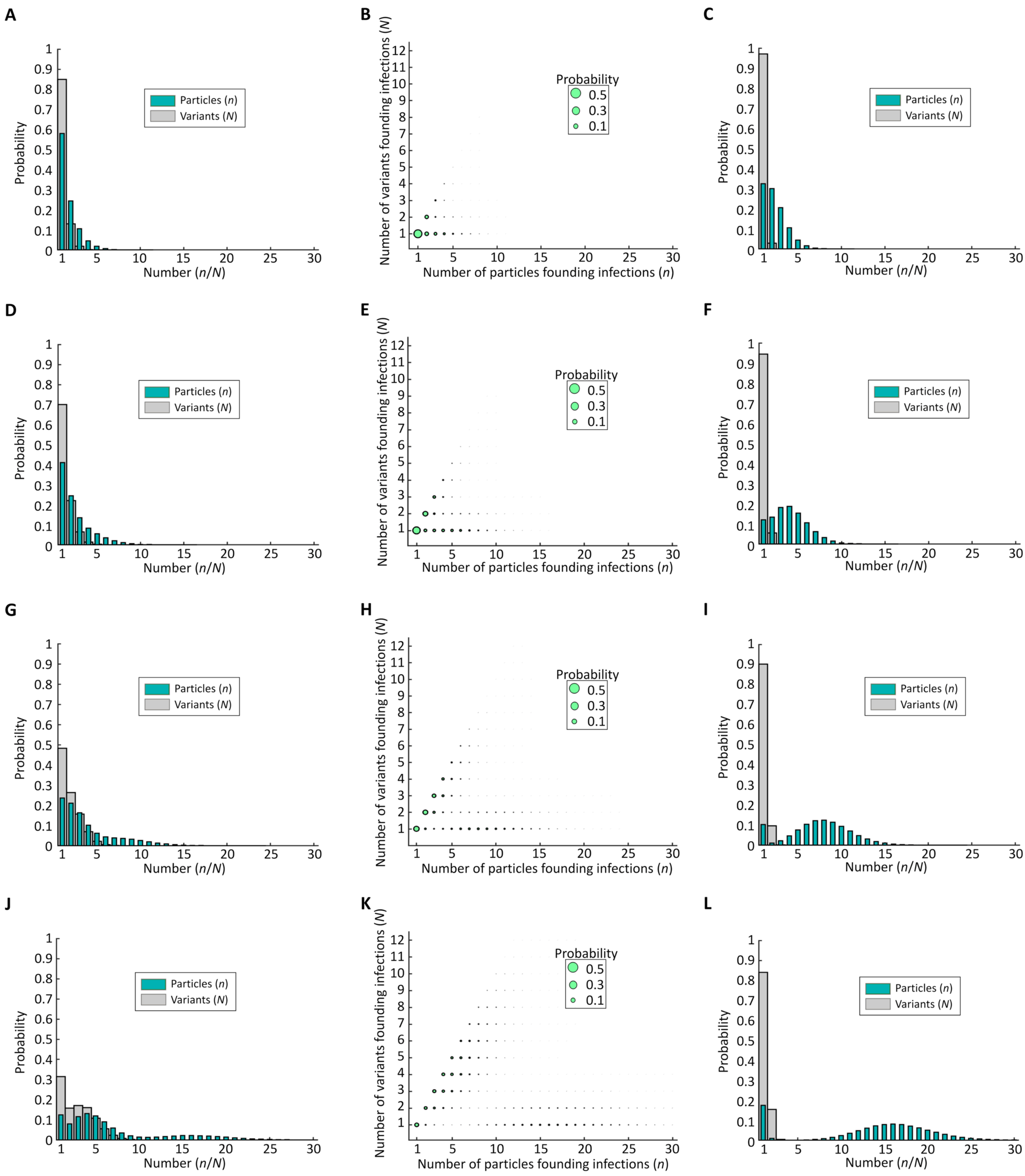

Figure S9. The distributions of the numbers of transmitted particles and variants for different values of the per-particle transmission probability (p). (A) The distributions of the numbers of particles (teal) and numbers of distinct variants (grey) founding new infections when the standard value of p in the no selection case is multiplied by factor 0.5. (B) The joint distribution of the numbers of particles and variants founding new infections when the standard value of p in the no selection case is multiplied by factor 0.5. (C) The distributions of the numbers of particles (teal) and numbers of distinct variants (grey) founding new infections in the population when the standard value of p in the no selection case is multiplied by factor 0.5, for donors in early infection only (infected for less than two years). (D)-(F), (G)-(I) and (J)-(L)

are figures analogous to A-C for factors 1, 2 and 4 respectively. We note that, by varying *p*, we are also testing the robustness of our results to the assumption that 30% of new infections are founded by multiple variants. For example, in panel A, 14% of infections are founded by multiple variants. The parameter *f* characterising the proportion of the time that the environment is appropriate for transmission could then be varied so that the per-act transmission probability is 0.003, but this would not alter the results in panels A-C which are conditional on transmission occurring. Parameter values: variant distribution parameter values are given in Table 1 of Text S1, and transmission parameter values are the same as in the no selection case given in Table 2 of Text S1 but with *p* amended as described above.

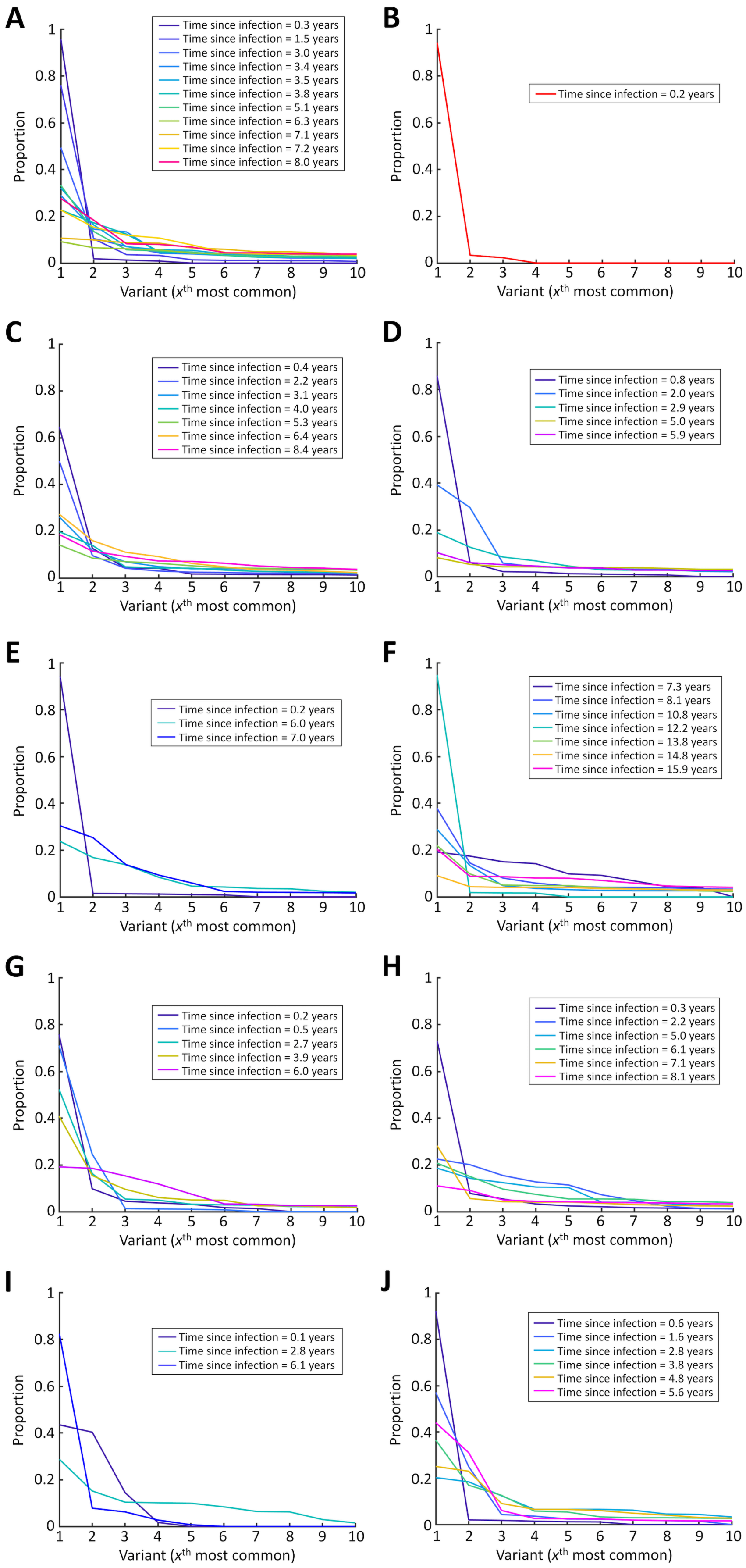

Figure S10. Representation of data showing the distribution of variants in the ten infected individuals during the course of untreated infection. All data are for integrase. Each panel corresponds to a different individual, with each line representing a different time of sampling in that individual. The *x*-axis represents the xth most common variant at

the time of sampling. Note that the xth most common variant at one time point does not necessary correspond to the xth most common variant at another time point. These data are obtained as described in Materials and Methods.

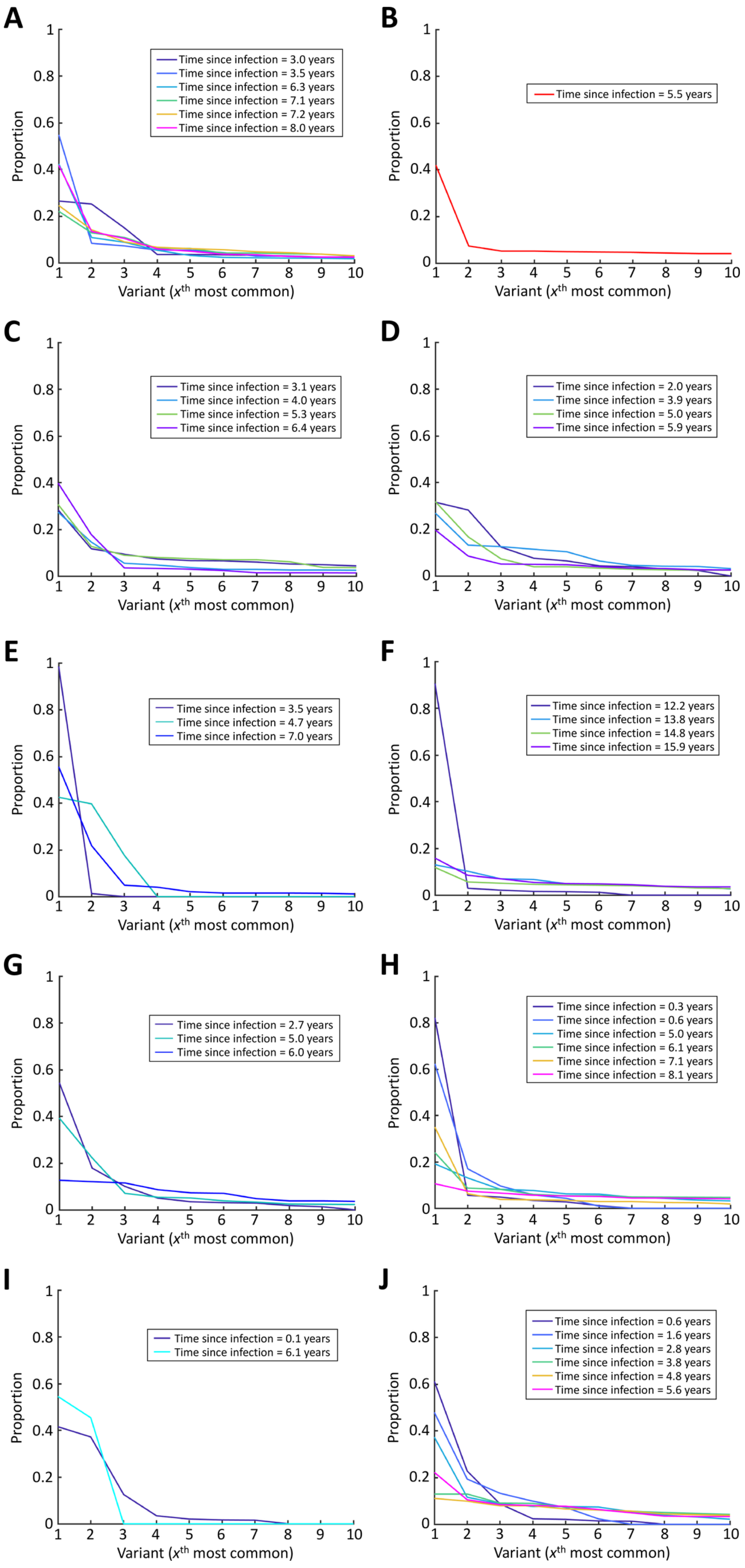

Figure S11. Representation of data showing the distribution of variants in the ten infected individuals during the course of untreated infection. All data are for p24. Each panel corresponds to a different individual, with each line representing a different time of sampling in that individual. The *x*-axis represents the xth most common variant at the

time of sampling. Note that the xth most common variant at one time point does not necessary correspond to the xth most common variant at another time point. These data are obtained as described in Materials and Methods.

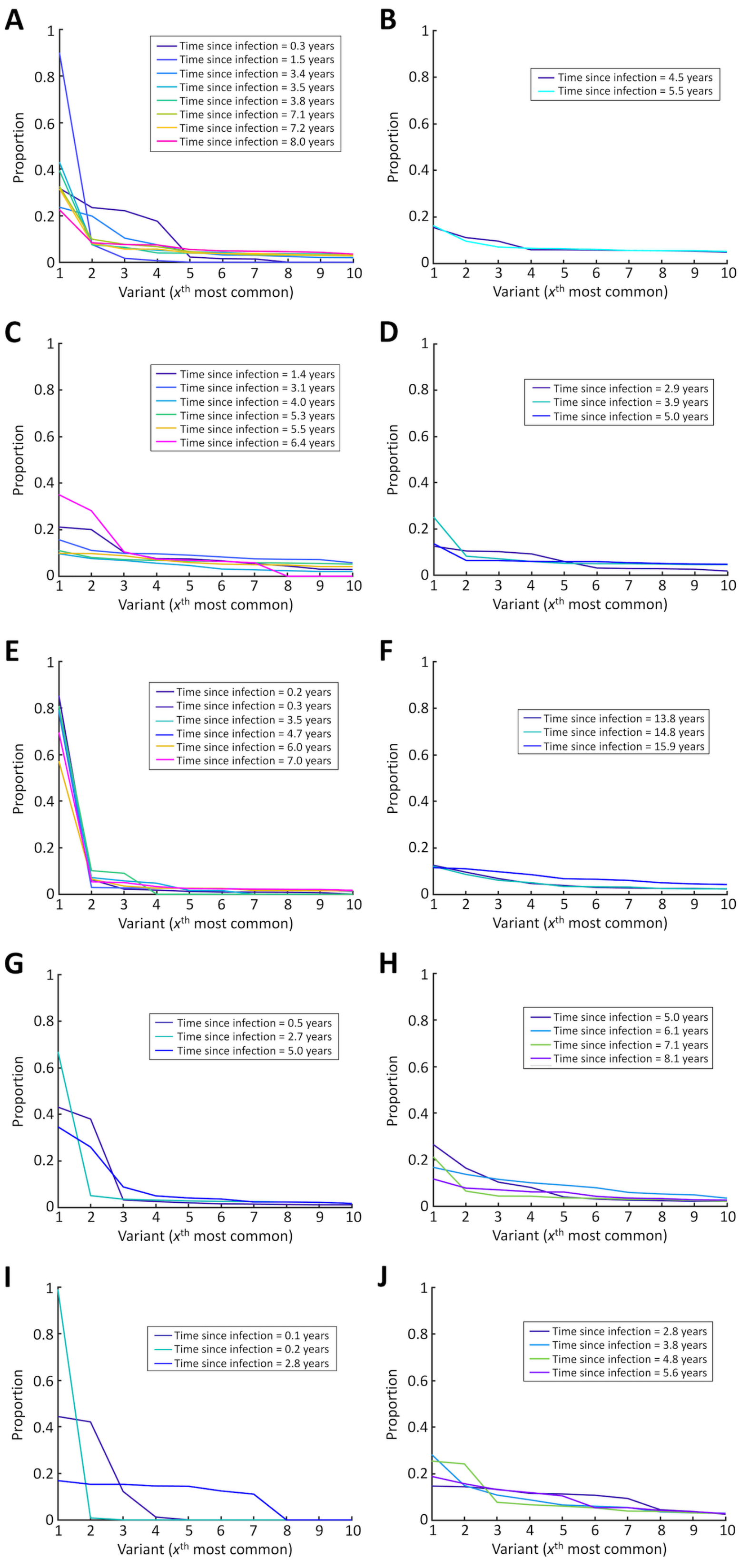

Figure S12. Representation of data showing the distribution of variants in the ten individuals during the course of untreated infection. All data are for nef. Each panel corresponds to a different individual, with each line representing a different time of sampling in that individual. The *x*-axis represents the xth most common variant at the time of sampling. Note that the xth most common variant at one time point does not necessary correspond to the xth most common variant at another time point. These data are obtained as described in Materials and Methods.
