# Supplementary material for: Link between the numbers of particles and variants founding new HIV-1 infections depends on the timing of transmission
Source: Virus Evol. 2019 Jan 30;5(1):vey038. doi: 10.1093/ve/vey038 (PMC6354028; doi:10.1093/ve/vey038)
Supplement: Supplementary Data [file vey038_supp.zip › supplementaryText.pdf]

Link between the numbers of particles and variants founding new HIV-1 infections depends on the timing of transmission

Text S1. Supporting Information

R.N. Thompson[1,2,3,*], C. Wymant[4], R.A. Spriggs[5], J. Raghwani[1,4], C. Fraser[4] and K.A. Lythgoe[1,4,*]

[1]Department of Zoology, University of Oxford, South Parks Road, Oxford OX1 3PS, UK

[2]Mathematical Institute, University of Oxford, Andrew Wiles Building, Radcliffe Observatory Quarter, Oxford OX2 6GG, UK

[3]Christ Church, University of Oxford, St Aldates, Oxford OX1 3DP, UK

[4]Big Data Institute, Li Ka Shing Centre for Health Information and Discovery, Nuffield Department of Medicine, University of Oxford, Oxford OX3 7FZ, UK

[5]Department of Plant Sciences, University of Cambridge, Downing Street, Cambridge CB2 3EA, UK

*Correspondence to: robin.thompson@chch.ox.ac.uk, katrina.lythgoe@bdi.ox.ac.uk

1. **Variant diversity in donors**
2. **Viral load profiles**
3. **Binomial models of transmission**
4. **Reconciling models of transmission with population-level data**
5. **Multiple variants founding infections in donors**
6. **Relationship between viral load and number of particles available for transmission**

1. Variant diversity in donors

In the main text, we described how the changing distribution of variants in donors during their courses of infection was estimated from next-generation sequencing data (Zanini et al. 2015). The best fitting parameter values for each of the models that we considered are given in the table below (Table 1 of Text S1).

Gene region	Strength of selection (α_s)	Model	δ estimate (standard error)	η estimate (standard error)	AIC relative to best fitting model for these data	Figures
Integrase	0	Gamma	0.417 (0.158)	0.563 (0.140)	0	3A, 4A, 4C, 5A, 5C, S2A-D, S3, S5A-C, S6, S7, S9, Fig 1 (Text S1), Fig 2 (Text S1), Fig 3 (Text S1), Fig 4 (Text S1), Fig 5 (Text S1)
Integrase	0	Pareto	1.09 (0.243)	N/A	+ 1058	N/A
Integrase	0	Exponential	1.53 (0.272)	N/A	+ 1605	N/A
p24	0	Gamma	0.502 (0.191)	0.439 (0.0942)	0	S1A-D, S4A-C
p24	0	Pareto	7.97 (4.42)	N/A	+ 377	N/A
p24	0	Exponential	1.56 (0.626)	N/A	+ 913	N/A
nef	0	Gamma	0.244 (0.166)	0.304 (0.0900)	0	S1E-H, S4D-F
nef	0	Pareto	0.813 (0.222)	N/A	+ 478	N/A
nef	0	Exponential	2.50 (0.546)	N/A	+ 723	N/A

Integrase	1	Gamma	0.301 (0.103)	0.779 (0.288)	0	S2E-H, S5D-F
Integrase	2	Exponential	6.18 (1.35)	N/A	0	S2I-L, S5G-I
Integrase	3	Exponential	9.65 (1.82)	N/A	0	3B, 4B, 5B, S2M-P, S5J-L

Table 1. Results of model fits to next-generation sequencing data to characterise the distribution of variants in donors throughout their courses of infection. Best fitting models for each dataset are those with the lowest AIC values – for example, for integrase data with no selection, the best fitting model is the gamma distribution.

2. Viral load profiles

To characterise the viral loads of infected individuals in the population, we follow a modelling approach used previously (Fraser et al. 2007). The infectious period of each infected individual is divided into three sub-periods: primary, chronic, and pre-AIDS. The viral loads in primary and pre-AIDS infection, and the length of these periods, are assumed to be the same for all donors in the population.

Primary infection

In the primary stage, which lasts $\tau_p = 0.24$ years, the assumed viral load for each individual is $V_p = 8.7 \times 10^7$ viral particles per millilitre.

Chronic infection

During the chronic stage of infection, the viral load, V_c, is fixed at set point (the set-point viral load, or SPVL), which varies by several orders of magnitude between different individuals (Henrard et al. 1995). The fraction of individuals at seroconversion (the point at which HIV-1 antibodies develop and become detectable) with each log(SPVL), v_c, in the population at any given time is described by

$$g_v(v_c) = \frac{2}{\sigma} z\left(\frac{v_c - \mu}{\sigma}\right) Z\left(\frac{\alpha(v_c - \mu)}{\sigma}\right),$$

where z and Z are the probability density function and cumulative distribution function of the standard normal distribution. We use published maximum-likelihood

estimates (Fraser et al. 2007), so that the mean of the distribution of log(SPVL) values is

$$\mu + \frac{2\sigma\alpha}{\sqrt{2\pi(1+\alpha^2)}} = 4.74,$$

and the standard deviation is

$$\sigma\sqrt{1 - \frac{2\alpha^2}{\pi(1+\alpha^2)}} = 0.78,$$

where α = -3.55, between minimum and maximum SPVL values of $V^{\min} = 10^2$ viral particles per millilitre and $V^{\max} = 10^7$ viral particles per millilitre.

However, rather than the proportion of *seroconverters* with each SPVL in the population, the quantity of interest is instead the proportion of *all infected individuals* that have each SPVL when they are in the chronic phase of infection (including individuals currently in the primary or pre-AIDS phases), $g(V)$. This distribution is obtained by weighting $g_V(v_c)$ by the relative lengths of the infectious periods of individuals with each SPVL and renormalising the resulting distribution. In doing this, we assume that the proportion of individuals with each SPVL is at equilibrium. This distribution is shown in Fig 2A in the main text.

The length of the chronic period is

$$\tau_c(V) = \frac{D_{\max} D_{50}^{D_k}}{V^{D_k} + D_{50}^{D_k}}$$

years, where $D_{\max}$ = 25.4 years, D_{50} = 3,058 copies per millilitre and D_k = 0.41. Consequently, higher SPVLs correspond to shorter chronic periods than lower SPVLs.

Pre-AIDS infection

The pre-AIDS stage of infection lasts τ_a = 0.75 years, and the viral load in this phase is assumed to be $V_a = 2.4 \times 10^7$ viral particles per millilitre for each individual.

3. Binomial models of transmission

Simple assumptions that could be made when modelling transmission are that each particle in an infected individual is transmitted in any given sex act with a fixed probability, *p*, say, and that each particle is transmitted independently of any other particle. As we illustrate here, this simple binomial model alone is inconsistent with real-world observations. In our analyses, we therefore adjust this model of transmission so that infections can only occur when environmental conditions are appropriate.

For an individual with *v* particles in the genital tract available for transmission, where each particle has an independent probability *p* of being transmitted during a single act, a simple binomial model is:

$$P_n = \text{Prob}(n \text{ particles transmitted}) = \binom{v}{n} p^n (1-p)^{v-n}.$$

Then

$$\begin{aligned} \text{Prob}(\text{transmission}) &= 1 - P_0, \\ &= 1 - (1-p)^v \\ &\approx vp, \end{aligned}$$

where we assume that *v* is large and *p* is small. The probability of transmission in real populations is small (around three out of every 1000 potential transmission acts – Boily et al. 2009), and hence *vp* must be small.

Then

$$\begin{aligned} \text{Prob}(\text{multi-particle transmission} \mid \text{transmission}) &= \frac{\text{Prob}(\text{multi-particle transmission})}{\text{Prob}(\text{transmission})}, \\ &= \frac{1 - P_0 - P_1}{1 - P_0}, \\ &= 1 - \frac{P_1}{1 - P_0}, \\ &= 1 - \frac{vp(1-p)^{v-1}}{1 - (1-p)^v}, \end{aligned}$$

$$= 1 - \frac{vp(1-p)^{v}(1-p)^{-1}}{1-\ (1-p)^{v}}.$$

Following Taylor expansion, this reduces to

$$\text{Prob}(\text{multi-particle transmission} \mid \text{transmission}) \approx \frac{vp}{2}.$$

This quantity is small, since we previously found *vp* to be small. Since transmission with multiple variants requires transmission with multiple particles, and this probability is small, then multi-variant transmission must also be rare. However, this is not the case in reality: around 30% of new infections are initiated with multiple variants (see e.g. Keele et al. 2008; Abrahams et al. 2008; Tully et al. 2016). Hence, the simplest possible binomial transmission models are not consistent with data observed in real populations.

In this subsection, we have only considered transmission from a single infected individual at a single time. If the complex relationships between viral load, viral diversity and duration of infection – which vary between individuals in a population – are included in a binomial model, we find the same result. To show this, we consider the transmission model in the main text but with $f = 1$ (analogous to a simple binomial model averaged over the entire population of donors): rare transmission again implies that new infections are almost always initiated by only single particles, and therefore only single variants (Figure 1 of Text S1).

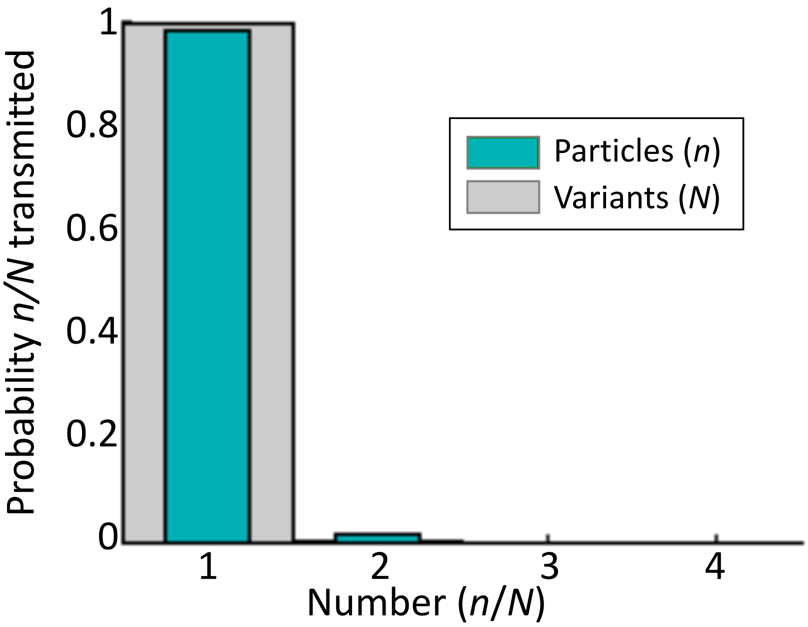

Figure 1 of Text S1. The numbers of particles and variants transmitted according to our model, but with parameters set so that there is a simple binomial assumption for the number of transmitted particles in each sex act. The environment is assumed to always be appropriate for transmission (f = 1), and the per-particle transmission probability p is chosen so that transmission occurs in 0.003 of transmission acts. The proportion of infections founded by multiple variants is then extremely low, contradicting what is observed in real populations. The variant distribution used for donors here corresponds to the no selection case, with transmission parameter values: $f = 1$, $p = 6 \times 10^{-10}$.

4. Reconciling models of transmission with population-level data

In heterosexual serodiscordant couples in low-income countries, transmission occurs in approximately three out of every 1000 sex acts and multiple variants found approximately 30% of new infections. We therefore set the value of the per-particle transmission probability (p) so that the probability of multiple variant transmission conditional on transmission occurring is 0.3. The distribution of particles and variants that are transmitted in sex acts in which transmissions are known to occur is then independent of the environmental suitability parameter f. However, the value of f can be chosen so that transmission occurs in three out of every 1000 transmission acts. The pairs of values (p, f) that match these population-level data (i.e. per-act transmission probability of 0.003 and probability of multi-variant transmission conditional on transmission of 0.3) in each of cases that we consider are shown in Table 2 of Text S1.

**Description**	***p***	***f***	**Relevant figures**
Integrase, Selection coefficient $\alpha_s = 0$	4.715×10^{-8}	0.029	4A, 4C, 5A, 5C, S5A-C, S6, S7, S9, Fig 2 (Text S1), Fig 3 (Text S1)
p24, $\alpha_s = 0$	4.34×10^{-8}	0.0296	S4A-C
nef, $\alpha_s = 0$	4.06×10^{-8}	0.031	S4D-F
Integrase, $\alpha_s = 1$	5.16×10^{-8}	0.028	S5D-F
Integrase, $\alpha_s = 2$	7.99×10^{-8}	0.0246	S5G-I
Integrase, $\alpha_s = 3$	1.215×10^{-7}	0.0222	4B, 5B, S5J-L
High variant diversity	4.36×10^{-8}	0.03	S8E-G
Low variant diversity	5.56×10^{-8}	0.027	S8L-N
Simple binomial model (environment always conducive)	4.715×10^{-8}	1	Fig 1 (Text S1)
Number of particles in genital tract lower than viral load per millilitre	9.4×10^{-8}	0.029	Fig 4A-C (Text S1)
Number of particles in genital tract higher than viral load per millilitre	1.18×10^{-8}	0.029	Fig 4D-F (Text S1)

Table 2. Values of the per-particle transmission probability (*p*) and the proportion of the time the environment is suitable for transmission (*f*) used in this manuscript. In most of our analyses, these values were set so that the per-act transmission probability is 0.003 and the probability of multi-variant transmission conditional on successful transmission is 0.3 in the absence of treatment.

5. Multiple variants founding infections in donors

In the main text, we described the fitting of distributions to estimate the proportion of each variant in donors throughout their courses of infection. Since model fits were obtained using data from ten infected individuals, the majority of whom were likely to have been infected by single variants initially (Puller et al. 2017), the fitted distributions suggest only a single variant in each donor initially. However, some donors in real populations will themselves have been infected by multiple variants. We therefore conduct a supplementary analysis to examine the numbers of variants expected to be transmitted from donors who were themselves instead assumed to be infected by multiple variants. Little is known about the viral dynamics through the course of infection in individuals infected by multiple variants from the same donor, with the notable exception of five individuals followed during the first few weeks of infection (Kijak et al. 2017). We therefore assumed that within-host lineages deriving from two different T/F variants evolved independently and remained at equal proportion during infection (for an example in which the donor was infected by two variants, see Fig 2E-H of Text S1). In other words, the diversity of variants within a host infected by two variants was changed from the fitted distribution $h(x,t)$ for x = 1,2,…N_s in the main text to $h_2(x,t) = h(\lceil x/2 \rceil, t)$, for x = 1,2,…2N_s, where the expression $\lceil x/2 \rceil$ represents the smallest integer greater than or equal to $x/2$. This is an extreme assumption, since within-host lineages may not evolve independently, and diversity may sometimes decrease during infection in some donors (Puller et al. 2017).

Under our assumptions, the number of variants that hosts initially infected with a pre-specified number of variants are likely to go on to transmit can then be derived (Fig 3 of Text S1). For example, for donors initially infected with two variants, this corresponds to the expression in the Materials and Methods in the main text for $\text{Prob}(N \text{ variants transmitted})$, but with the variant distribution $h(x,t)$ replaced with $h_2(x,t)$,

$\text{Prob}(N \text{ variants transmitted}) =$

$$f \sum_{n_c=n_c^{\min}}^{n_c^{\max}} g(n_c) \Bigg(\int_0^{\tau_p} \sum_{n=N}^{n_p} \text{Prob(Transmit } N \text{ variants} \mid \text{variant distribution } h_2(x,\tau), \text{transmit } n \text{ particles)} \frac{1}{\tau_p+\tau_c(n_c)+\tau_a} \binom{n_p}{n} p^n (1-p)^{n_p-n} \, \mathrm{d}\tau +$$

$$\int_{\tau_p}^{\tau_p+\tau_c(n_c)} \sum_{n=N}^{n_c} \text{Prob(Transmit } N \text{ variants} \mid \text{variant distribution } h_2(x,\tau), \text{transmit } n \text{ particles)} \frac{1}{\tau_p+\tau_c(n_c)+\tau_a} \binom{n_c}{n} p^n (1-p)^{n_c-n} \, \mathrm{d}\tau +$$

$$\int_{\tau_p+\tau_c(n_c)}^{\tau_p+\tau_c(n_c)+\tau_a} \sum_{n=N}^{n_a} \text{Prob(Transmit } N \text{ variants} \mid \text{variant distribution } h_2(x,\tau), \text{transmit } n \text{ particles)} \frac{1}{\tau_p+\tau_c(n_c)+\tau_a} \binom{n_a}{n} p^n (1-p)^{n_a-n} \, \mathrm{d}\tau \Bigg). \qquad \text{(S1)}$$

We find that hosts initially infected with multiple variants are more likely to go on to transmit multiple variants. However, this result is strongest if the host is in very early infection when they transmit to another host: by late infection, the virus has diversified so that the viral profile of a donor infected with a single T/F variant is similar to a donor infected with multiple T/F variants. As a result, if a host transmits in late infection, the initial number of variants that the donor was infected with will not drastically change the number of variants that they are likely to go on to transmit.

We repeat the analysis from the main text of the number of particles and variants founding new infections, instead in a population in which 30% of donors were themselves infected by two variants, and the rest infected by a single variant. (Fig 2I-K of Text S1). This involves conditioning on whether each new infection arises from a donor who was infected by one variant or from a donor who was infected by two variants. For example, the equation for the number of variants transmitted in each transmission act becomes

$$\begin{aligned} &\text{Prob}(N \text{ variants transmitted}) \\ &= 0.7 \times \text{Prob}(N \text{ variants transmitted} \mid \text{donor initially infected with one variant}) \\ &+ 0.3 \times \text{Prob}(N \text{ variants transmitted} \mid \text{donor initially infected with two variants}). \end{aligned}$$

The expression $\text{Prob}(N \text{ variants transmitted} \mid \text{donor initially infected with one variant})$ is simply the expression for the distribution of transmitted variants in the main text. The term $\text{Prob}(N \text{ variants transmitted} \mid \text{donor initially infected with two variants})$ is given by equation (S1) above. The entire expression for Prob(N variants transmitted)

was then normalised (for N = 1,2,3,…), to give a valid probability distribution for the number of transmitted variants conditional on transmission occurring.

Our main result is that, as before, the link between the numbers of T/F particles and T/F variants depends on the timing of transmission, with infections deriving from donors in early infection again more likely to be founded by a large number of particles but only a small number of variants.

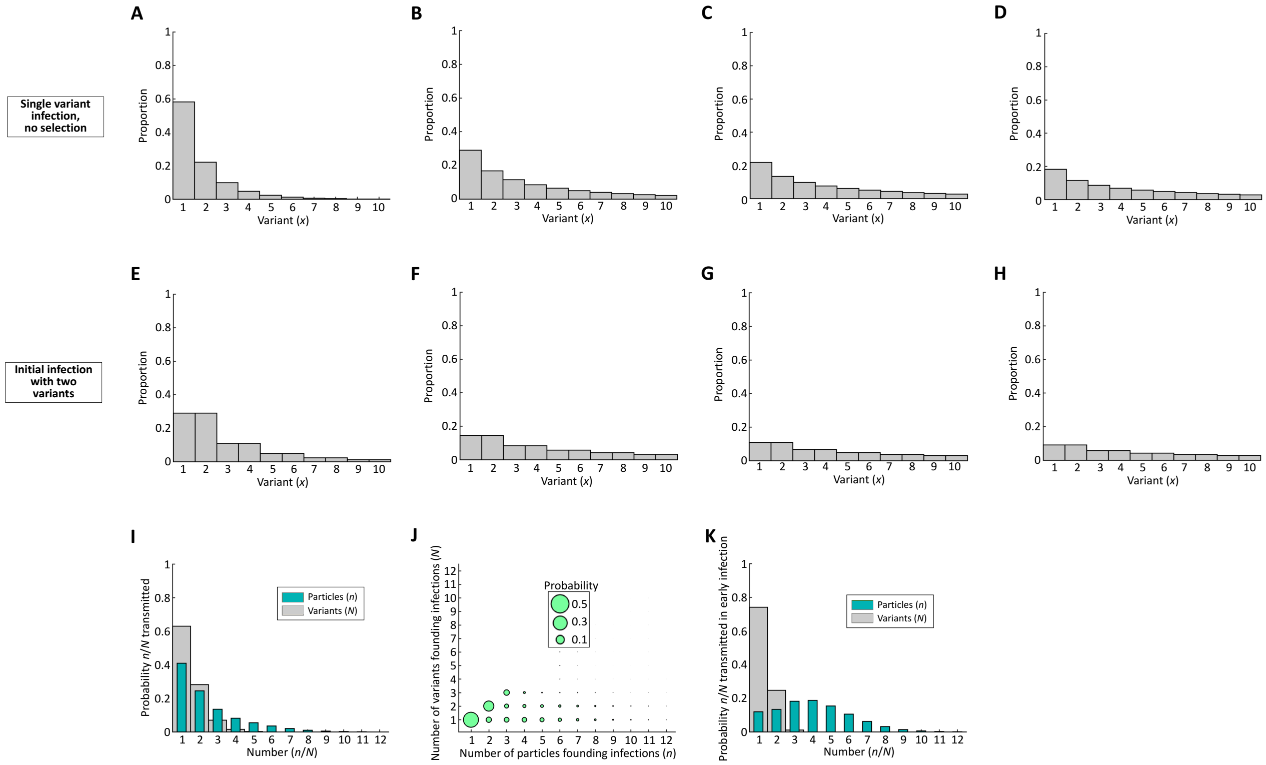

Figure 2 of Text S1. The distributions of transmitted particles and variants in a population in which 70% of donors were themselves infected by one variant and 30% of donors were infected by two variants. The distribution of variants in donors themselves infected by a single variant after: (A) 1 year; (B) 4 years; (C) 7 years; (D) 10 years. (E)-(H) The distribution of variants in donors themselves infected by two variants at the same timepoints as A-D. The x-axis represents the xth most common variant at each of the above timepoints. (I) The distributions of the numbers of particles (teal) and numbers of distinct variants (grey) founding new infections in a population in which 70% of infections are from donors are infected by a single variant and 30% of infections are from donors infected by two variants. (J) The joint probability distribution of the numbers of particles and variants founding new infections for the same population as I; the area of each circle is proportional to the probability that the number of particles on the x-axis and number of variants on the y-axis are transmitted in a single successful transmission act. (K) Identical figure to I, for donors in early infection only (infected for less than two years). Parameter values shown in Tables 1 and 2 of Text S1.

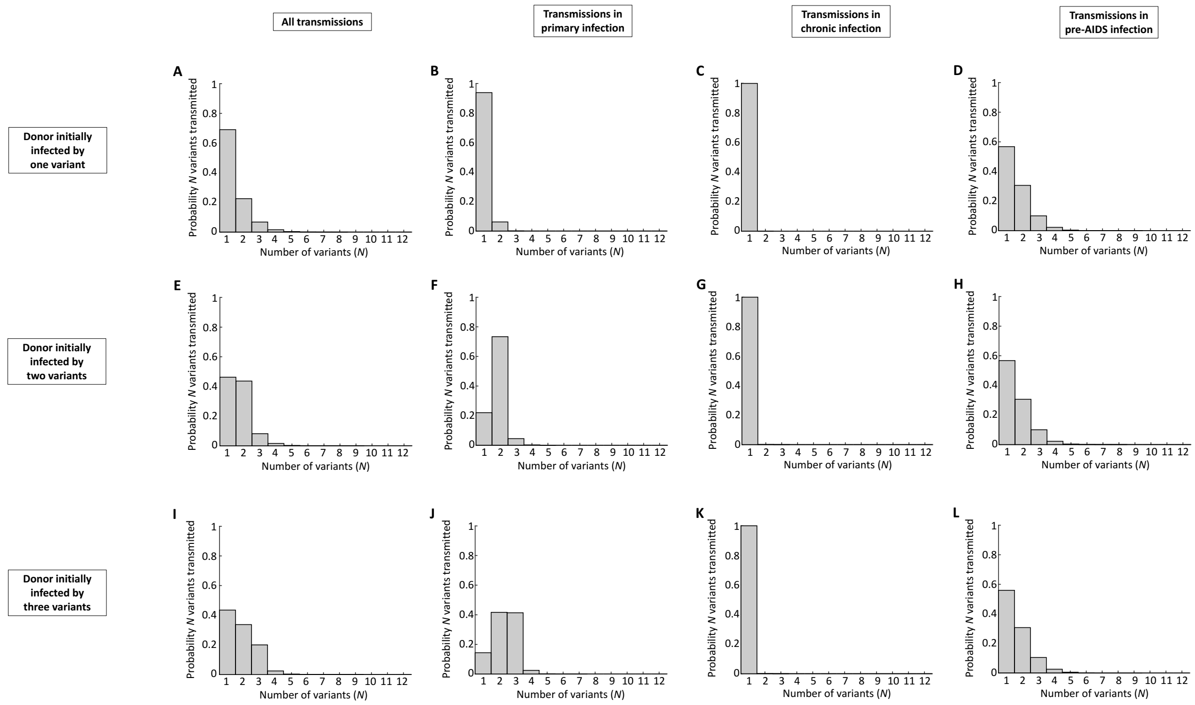

Figure 3 of Text S1. The numbers of variants expected to be transmitted by a donor, for different numbers of T/F variants in that donor. The first column represents all transmissions. The second, third and fourth columns represent transmissions in primary, chronic and pre-AIDS infection, respectively. The distribution of the number of transmitted variants are shown for: (A)-(D) Donor infected by a single variant initially. (E)-(H) Donor infected by two variants initially. (I)-(L) Donor infected by three variants initially. The donor considered here has intermediate viral load, with $n_c = 10^{4.5}$ particles available for transmission during chronic infection. Parameter value: $p = 4.715 \times 10^{-8}$. The lineages from each initial variant in the donor are assumed to evolve independently and generate different variants. The relative frequency of each of the initial infecting variants is assumed to be equal, and these variants each evolve according to the distributions shown in the left column of Fig 3 of the main text.

6. Relationship between viral load and number of particles available for transmission

In the analysis in the main text, we assumed that the number of particles available for transmission in the genital tract of each donor is proportional to the viral load, with constant of proportionality $k = 1$. For example, if the viral load is $V = 100{,}000$ viral particles per millilitre, then we assume that 100,000 particles are available for transmission. However, the constant of proportionality could in principle take other values, and so we conduct a supplementary analysis considering different values of k (0.5 and 4). For each value of k, the model is reparameterised to fit the population-

level data (i.e. per-act transmission probability of 0.003 and probability of transmitting multiple variants of 0.3), and the distributions of transmitted particles and variants are approximately unchanged (Fig 4 of Text S1).

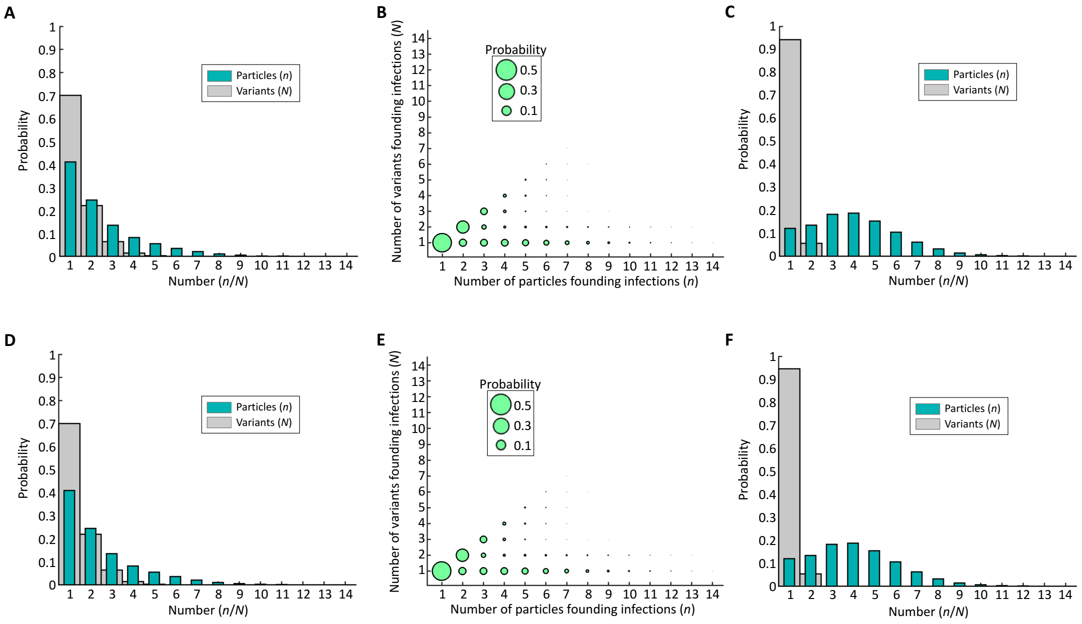

Figure 4 of Text S1. The effect of the numbers of particles available for transmission in donors' genital tracts on the numbers of particles and viral variants that found new infections in the population. (A) The numbers of particles (green) and variants (grey) founding new infections when the number of particles in the genital tract is smaller than the viral load per millilitre (k = 0.5). (B) The joint probability distribution of the numbers of transmitted particles and variants when the number of particles in the genital tract is smaller than the viral load per millilitre (k = 0.5). (C) The distributions of the numbers of particles (green) and numbers of distinct variants (grey) founding new infections in the population, from donors in early infection only (infected for less than two years), when the number of particles in the genital tract is smaller than the viral load per millilitre (k = 0.5). Panels D-F are equivalent to A-C, when the number of particles in the genital tract is larger than the viral load per millilitre (k = 4). In B and E, circle areas are proportional to the probabilities that they represent. Parameter values: variant distribution parameter values are given in Table 1 of Text S1, and transmission parameter values are given in Table 2 of Text S1.

The reason that the value of k has little effect on the distributions of transmitted particles and variants is that these quantities depend only on the product of k and the per-particle probability of transmission, p, rather than the individual values of these parameters. When the value of p is fitted so that the per-act transmission probability is 0.003, then higher values of k simply corresponds to choosing lower values of p, so

that the product *kp* remains approximately fixed. We demonstrate that the distribution of transmitted particles – and therefore also the distribution of transmitted variants – depends approximately on the product of *k* and *p*, and not the individual values, here.

In the scenario in the main text, which corresponds to the case in which *k* = 1 so that the number of particles available for transmission in each donor's genital tract is equal to the viral load per millilitre (i.e. $n_p = V_p$, $n_c = V_c$ and $n_a = V_a$), the distribution for the numbers of transmitted particles was given by

$$\text{Prob}(n \text{ particles transmitted}) =$$

$$f \sum_{n_c=n_c^{\min}}^{n_c^{\max}} g(n_c) \left(\frac{\tau_p}{\tau_p + \tau_c(n_c) + \tau_a} \binom{n_p}{n} p^n (1-p)^{n_p-n} + \frac{\tau_c(n_c)}{\tau_p + \tau_c(n_c) + \tau_a} \binom{n_c}{n} p^n (1-p)^{n_c-n} + \frac{\tau_a}{\tau_p + \tau_c(n_c) + \tau_a} \binom{n_a}{n} p^n (1-p)^{n_a-n} \right).$$

If instead, however, the numbers of particles available for transmission in primary, chronic and pre-AIDS infection are assumed to be $n_p = kV_p$, $n_c = kV_c$ (for the different possible values of V_c between infected individuals, which range between $V_c^{\min}$ and $V_c^{\max}$) and $n_a = kV_a$, respectively, then this expression is altered to give

$$\text{Prob}(n \text{ particles transmitted}) =$$

$$f \sum_{V_c=V_c^{\min}}^{V_c^{\max}} g(V_c) \left(\frac{\tau_p}{\tau_p + \tau_c(V_c) + \tau_a} \binom{kV_p}{n} p^n (1-p)^{kV_p-n} + \frac{\tau_c(V_c)}{\tau_p + \tau_c(V_c) + \tau_a} \binom{kV_c}{n} p^n (1-p)^{kV_c-n} + \frac{\tau_a}{\tau_p + \tau_c(V_c) + \tau_a} \binom{kV_a}{n} p^n (1-p)^{kV_a-n} \right).$$

If kV_p, kV_c and kV_a are large, and *p* is assumed to be very small (so that $kV_p p$, $kV_c p$ and $kV_a p$ are not large), then the binomial distributions can be approximated instead by Poisson distributions,

$$\text{Prob}(n \text{ particles transmitted}) \approx$$

$$f \sum_{V_c=V_c^{\min}}^{V_c^{\max}} g(V_c) \left(\frac{\tau_p}{\tau_p + \tau_c(V_c) + \tau_a} \frac{(kV_p p)^n \exp(-kV_p p)}{n!} + \frac{\tau_c(V_c)}{\tau_p + \tau_c(V_c) + \tau_a} \frac{(kV_c p)^n \exp(-kV_c p)}{n!} + \frac{\tau_a}{\tau_p + \tau_c(V_c) + \tau_a} \frac{(kV_a p)^n \exp(-kV_a p)}{n!} \right).$$

The number of particles transmitted in a potential transmission act therefore depends only on the product *kp* rather than the individual values of these parameters. The distribution of transmitted variants depends on this expression and the distribution of variants in the donor (which is independent of *k* and *p*). Consequently, the distribution of transmitted variants also depends only on the product *kp* rather than the individual values.

If the value of *k* is increased, then refitting the model so that Prob(transmission) = 0.003 corresponds to lowering *p* by the same factor, so that *kp* remains fixed. As a result, the distributions for the probability of transmitting *n* particles and the probability of transmitting *N* variants also remain fixed. Consequently, our results are hardly affected by the constant of proportionality *k* representing the ratio of the viral load of a donor to the corresponding number of particles available for transmission in that donor's genital tract.

<u>References</u>

Abrahams MR, Anderson JA, Giorgi EE, Seoighe C, Mlisana K, Ping LH, Athreya GS, Treurnicht FK, Keele BF, Wood N, Salazar-Gonzalez JF. 2009. Quantitating the multiplicity of infection with human immunodeficiency virus type 1

subtype C reveals a non-poisson distribution of transmitted variants. *J Virol* **83**: 3556-3567.

Boily MC, Baggaley RF, Wang L, Masse B, White RG, Hayes RJ, Alary M. 2009. Heterosexual risk of HIV-1 infection per sexual act: systematic review and meta-analysis of observational studies. *Lancet Inf Dis* **9**: 118-129.

Fraser C, Hollingsworth TD, Chapman R, de Wolf F, Hanage WP. 2007. Variation in HIV-1 set-point viral load: epidemiological analysis and an evolutionary hypothesis. *Proc Natl Acad Sci* **104**: 17441-6.

Henrard DR, Phillips JF, Muenz LR, Blattner WA, Wiesner D, Eyster ME, Goedert JJ. 1995. Natural history of HIV-1 cell-free viremia. *JAMA* **274**: 554–558.

Keele BF, Giorgi EE, Salazar-Gonzalez JF, Decker JM, Pham KT, Salazar MG, Sun C, Grayson T, Wang S, Li H, Wei X. 2008. Identification and characterization of transmitted and early founder virus envelopes in primary HIV-1 infection. *Proc Natl Acad Sci* **105**: 7552–7557.

Kijak GH, Sanders-Buell E, Chenine AL, Eller MA, Goonetilleke N, Thomas R, Leviyang S, Harbolick EA, Bose M, Pham P, Oropeza C. 2017. Rare HIV-1 transmitted/founder lineages identified by deep viral sequencing contribute to rapid shifts in dominant quasispecies during acute and early infection. *PLoS Pathog* **13**: e1006510.

Puller V, Neher R, Albert J. Estimating time of HIV-1 infection from next-generation sequence diversity. *PLoS Comput Biol* **13**: e1005775.

Tully DC, Ogilvie CB, Batorsky RE, Bean DJ, Power KA, Ghebremichael M, Bedard HE, Gladden AD, Seese AM, Amero MA, Lane K. 2016. Differences in the selection bottleneck between modes of sexual transmission influence the genetic composition of the HIV-1 founder virus. *PLoS Pathog* **12**: e1005619.

Zanini F, Brodin J, Thebo L, Lanz C, Bratt G, Albert J, Neher RA. 2015. Population genomics of intrapatient HIV-1 evolution. *eLife* **4**: e112.
